# Prevalence and trends of sensitisation to aeroallergens in patients with allergic rhinitis in Guangzhou, China: a 10-year retrospective study

**DOI:** 10.1136/bmjopen-2016-011085

**Published:** 2016-05-16

**Authors:** Weihao Wang, Xuekun Huang, Zhuanggui Chen, Rui Zheng, Yulian Chen, Gehua Zhang, Qintai Yang

**Affiliations:** 1 Department of Otorhinolaryngology—Head and Neck Surgery, The Third Affiliated Hospital of Sun Yat-Sen University, Guangzhou, China; 2 Department of Pediatrics, The Third Affiliated Hospital of Sun Yat-Sen University, Guangzhou, China; 3 Department of Otorhinolaryngology—Head and Neck Surgery, The First Affiliated Hospital of Sun Yat-Sen University, Guangzhou, China

**Keywords:** allergic rhinitis, serum sIgE, house dust mite, pet allergens

## Abstract

**Objective:**

To investigate the prevalence and trends of sensitisation to common aeroallergens among outpatients with allergic rhinitis (AR) in Guangzhou, China, over the past decade.

**Design:**

A retrospective study; linear-by-linear association and simple linear regression were used to determine the trends in the prevalence of aeroallergen sensitisation.

**Setting:**

One grade-A hospital in Guangzhou, the largest city in southern China.

**Participants:**

A total of 5486 patients (2297 males and 2489 females) who visited the ear, nose and throat outpatient clinic, from January 2005 to December 2014, were enrolled. All patients who presented with nasal hyper-reactive symptoms and who completed serological allergy testing, measuring specific IgE (sIgE) in the serum, were included. Among them, 4085 participants (2269 males and 1816 females) were diagnosed as being patients with AR.

**Outcome measures:**

Prevalence and trends of sensitisation to various types of aeroallergens were assessed.

**Results:**

The overall prevalence of sIgE-mediated sensitisation to aeroallergens in these patients with AR were as follows: 84.4% for house dust mites (HDMs), 23.4% for pet allergens, 21.1% for cockroaches, 9.1% for mould allergens, 7.7% for tree pollen and 6.0% for weed pollen. When all patients with nasal hyper-reactivity were stratified by decade of age, increasing age was associated with a decrease in sIgE positivity by ∼5.13% (95% CI −7.28% to −2.98%, p<0.01). Within the past decade, the prevalence of sensitisation to pet allergens in patients with AR increased at an annual rate of 1.3% (95% CI 0.85% to 1.67%, p<0.01).

**Conclusions:**

This study demonstrated that HDMs comprised the most common aeroallergen in Guangzhou. The prevalence of sensitisation to aeroallergens decreased with increasing age. During the past decade, the prevalence of sensitisation to pet allergens showed an upward trend, suggesting an urgent need for its prevention and treatment.

**Strengths and limitations of this study**

- This is the first study investigating the trends in the prevalence over the last decade and specific IgE (sIgE)-mediated sensitisation profiles in patients with allergic rhinitis (AR) in Guangzhou, China.
- The study was conducted based on the results of serum sIgE assessment, a standard diagnostic test with high sensitivity and specificity for common aeroallergens.
- This is a retrospective clinical study. The patients' history records on the severity of AR symptoms and on the potential risk factors in association with allergen exposure are incomplete.
- The study was primarily a single-centre study. The prevalence and pattern of allergen sensitisation profiles in the general population in Guangzhou remain unknown.

## Introduction

Allergic rhinitis (AR) is the most common allergic respiratory disease, affecting 10–40% of individuals worldwide. AR can adversely affect the quality of life and impose a substantial burden on patients and society.^1^ ^2^ AR is an IgE-mediated type I nasal allergic disorder characterised by nasal hyper-reactivity symptoms, including nasal pruritus, sneezing, airflow obstruction and rhinorrhoea.^3^ It is crucial during diagnosis to distinguish the type of allergen causing AR symptoms and to identify an effective therapy. From the literature, the pattern of allergic sensitisation profiles varies in different countries and regions. In the UK, grass pollen was the most common aeroallergen, while cat allergens and cedar pollen were the most common aeroallergen for AR in Sweden and Japan, respectively.^4–6^ China is suffering from an increasing incidence of AR. A multicentre study performed by Li *et al*^7^ found that the prevalence of sensitisation to pollens and house dust mites (HDMs) was higher in the northern and southern regions, respectively. Recently, the global prevalence of AR has steadily increased, which may be attributed to urbanisation, resulting in subsequent environmental and lifestyle changes due to economic development.^8–10^ A study by Mehulić *et al*^11^ reported a significant change in the sensitisation to different aeroallergens in Croatia. However, there is a lack of such data in Guangzhou, the largest city in southern China. Thus, the aim of this study was to investigate the prevalence and trends of allergic sensitisation to common aeroallergens in patients with AR in Guangzhou, over the past decade.

## Materials and methods

### Study design and participants

We performed a retrospective study based on the results of serum-specific IgE (sIgE) tests from 5486 patients with nasal hyper-reactivity symptoms who attended the ear, nose and throat outpatient clinics at the Third Affiliated Hospital of Sun Yat-Sen University in Guangzhou, China, from January 2005 to December 2014. The inclusion criteria for enrolled participants were: (1) presence of nasal hyper-reactivity symptoms, including sneezing and a runny, itchy or blocked nose and (2) a completed serum sIgE test for common aeroallergens. Therefore, all patients with nasal hyper-reactivity symptoms were included in this study; 4085 patients with AR met the diagnostic criteria.^12^

The study patients were from a wide range of geographical area; they came from the following districts in Guangzhou: Tianhe (n=2269, 41.4%), Yuexiu (n=1048, 19.1%), Haizhu (n=735, 13.4%), Liwan (n=623, 11.4%), Baiyun (n=438, 8.0%), and Conghua and Zengcheng (n=373, 6.8%).

All participants signed informed consent forms.

### sIgE measurements

The Allergy Screen test (Mediwiss Analytic GmbH, Moers, Germany) was used to measure serum sIgE levels for common aeroallergens. The following six types of common aeroallergens were detected: HDMs (*Dermatophagoides pteronyssinus*), pet allergens (combination of dog hair and cat dander), cockroaches, moulds (mixture of *Penicillium notatum*, branch spore mildew, *Aspergillus fumigates* and *Alternaria*), tree pollens (combination of cypress, elm, phoenix tree, willow and cottonwood) and weed pollens (combination of short ragweed, mugwort, *Humulus scandens* and pigweed). The serum sIgE levels were calculated using a calibration curve and expressed as a concentration of international units per millilitre (IU/mL). In accordance with the manufacturer's protocols, the concentrations of sIgE were quantitatively ranked as follows: class 0, <0.35 IU/mL; class 1, ≥0.35 to 0.70 IU/mL; class 2, ≥0.70–3.5 IU/mL; class 3, ≥3.5–17.5 IU/mL; class 4, ≥17.5–50 IU/mL; class 5, ≥50–100 IU/mL; class 6, ≥100 IU/mL. Serum sIgE levels of ≥0.35 IU/mL (class 1 or above) were considered positive.

### Statistical analysis

The IBM-SPSS (V.20; SPSS Inc, Chicago, Illinois, USA) statistical software package was used for data processing and analysis. Linear-by-linear association was used to investigate whether or not the changing prevalence of aeroallergen sensitisation was characterised by a significant linear-by-linear association with years or increasing ages. And simple linear regression was used to analyse how the overall trends in the prevalence of sensitisation to aeroallergens occurred among age groups or year groups on the premise of the significant linear-by-linear association. Owing to ages not being normally distributed, we used the Mann-Whitney U test to compare the age difference between genders. A p<0.05 was considered statistically significant.

## Results

### Demographic data

Among the 5486 patients, 4085 participants were diagnosed as AR, 2269 males (55.5%) and 1816 females (44.5%), aged between 1 and 79 years (median age=23.0 years, IQR 10.0–32.0). The median ages were 19.0 years (IQR 8.5–30.0) for males and 26.0 years (IQR 15.0–33.0) for females. The age of sensitisation to aeroallergens in males was significantly younger than in females (p<0.01).

### Prevalence of sensitisation to aeroallergens in Guangzhou

Among the 4085 patients with AR, the prevalence rates of sensitisation to aeroallergens were as follows: 84.4% for HDMs, 23.4% for pet allergens (combination of dog hair and cat dander), 21.1% for cockroaches, 9.1% for mould allergens, 7.7% for mixed tree pollens and 6.0% for mixed weed pollen.

### Allergen sensitisation by age group

**Figure 1:**
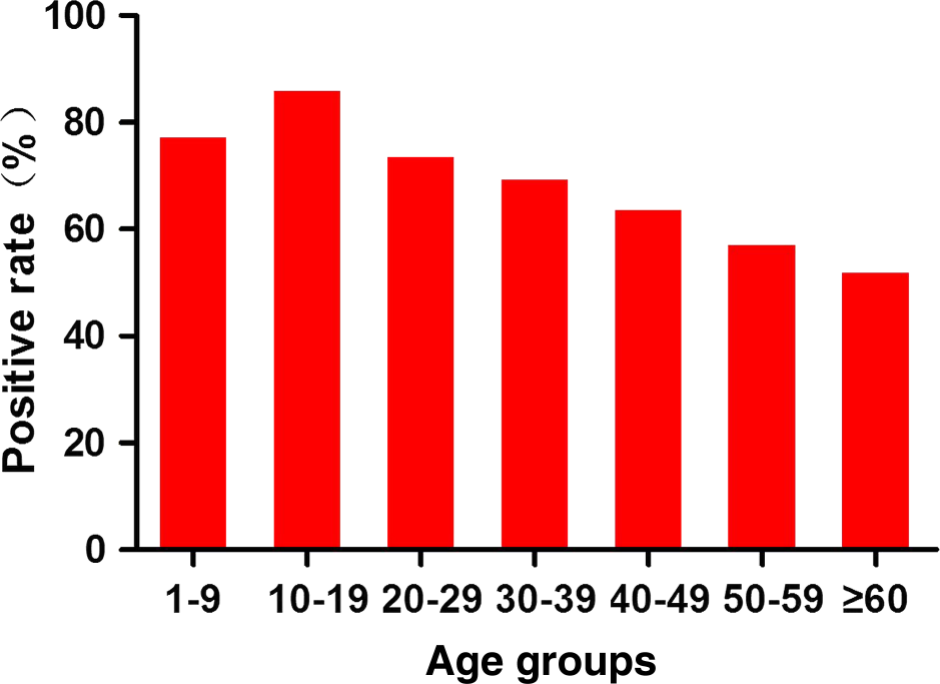
Age differences in the specific IgE-positive rates of sensitisation to at least one aeroallergens.

To further assess the impact of age on the prevalence of allergic sensitisation among the patients with nasal hyper-reactivity symptoms, we grouped them into the following age groups: 1–9, 10–19, 20–29, 30–39, 40–49, 50–59 and ≥60 years. The prevalence of allergic sensitisation to aeroallergens for each age group is shown in figure 1. The prevalence was the highest in the 10–19-year age group (86.6%) and the lowest in the ≥60 year age group (52.6%). The sIgE reactivity decreased as age increased, and with 10-year age increase the prevalence decreased by 5.13% (95% CI −7.28% to −2.98%, p<0.01).

The highest prevalence of allergic sensitisation to HDMs in patients with AR was in the age group of 10–19 years (91.4%), and this decreased with age (p<0.01). It decreased by 3.0% every 10 years after the age of 19 years (95% CI −5.6% to −0.45%, p<0.05). Sensitisation to pet allergens was higher in the 1–9-year age group (30.2%) than in the other age groups. The prevalence of sensitisation to weed pollen increased on average by 2.09% for every 10 years (95% CI 1.28% to 2.90%, p<0.05). Similarly, the prevalence of sensitisation to tree pollen showed an upward trend, increasing with age (p<0.001) at a rate of 1.36% every 10 years (95% CI 0.17% to 2.56%, p<0.05; table 1).

**Table 1:** The prevalence of sensitisation to each kind of aeroallergen in age groups

	HDMs	Pet allergens	Cockroaches	Mixed mould	Weed pollens	Tree pollens
1–9 years (n=948)	83.1% (788/948)	30.2% (286/948)	11.6% (110/948)	11.1% (105/948)	2.4% (23/948)	4.6% (44/948)
10–19 years (n=798)	91.4% (729/798)	25.1% (200/798)	18.5% (148/798)	8.9% (71/798)	3.8% (30/798)	5.1% (41/798)
20–29 years (n=1079)	84.2% (909/1079)	20.8% (224/1079)	28.1% (303/1079)	7.7% (83/1079)	8.0% (86/1079)	8.9% (96/1079)
30–39 years (n=786)	82.7% (650/786)	18.3% (144/786)	24.8% (195/786)	8.3% (65/786)	6.2% (49/786)	9.9% (78/786)
40–49 years (n=315)	83.2% (262/315)	19.4% (61/315)	23.2% (73/315)	8.9% (28/315)	10.5% (33/315)	10.2% (32/315)
50–59 years (n=108)	68.5% (74/108)	25.9% (28/108)	19.4% (21/108)	9.3% (10/108)	14.8% (16/108)	15.7% (17/108)
≥60 years (n=51)	70.6% (36/51)	21.6% (11/51)	21.6% (11/51)	15.7% (8/51)	13.7% (7/51)	9.8% (5/51)
p Value*	<0.05	<0.05	<0.05	0.18	<0.05	<0.05
p Value†	<0.01	0.30	0.40	0.31	<0.01	<0.05

*Linear-by-linear association.

†Simple linear regression.

HDM, house dust mite.

### Trends in the prevalence of sensitisation to aeroallergens over the past decade

**Figure 2:**
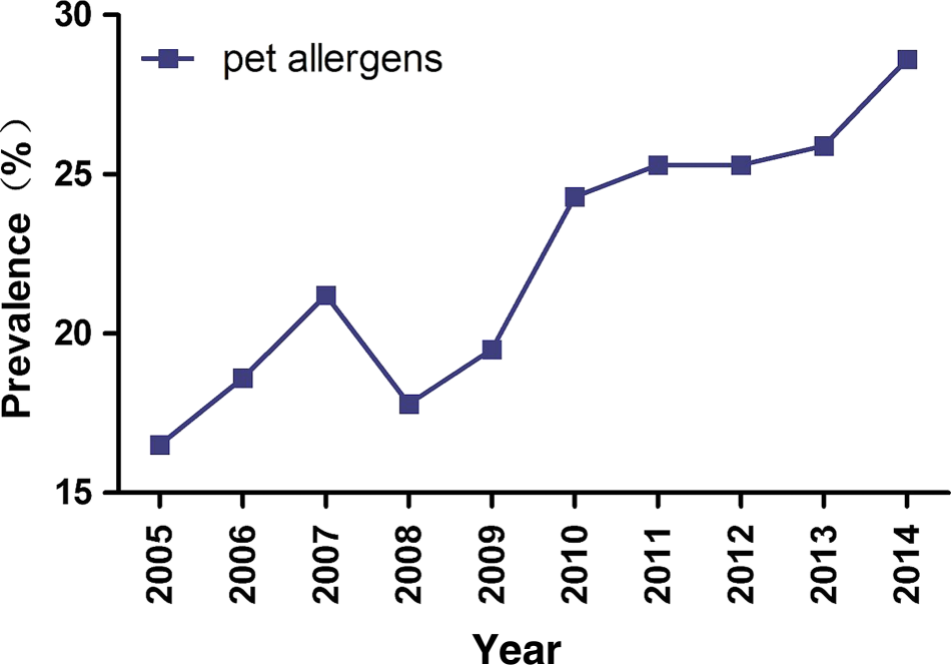
The significant uptrend of the prevalence of sensitisation to pet allergens in patients with allergic rhinitis from 2005 to 2014.

The prevalence rates of sensitisation to at least one aeroallergen from 2005 to 2014 were 69.7% (2005), 73.6% (2006), 75.1% (2007), 72.0% (2008), 75.7% (2009), 76.6% (2010), 74.1% (2011), 75.8% (2012), 75.9% (2013) and 74.0% (2014). During these 10 years, we did not observe a significant trend among the prevalence rates (p>0.05). There were no significant differences in the prevalence of sensitisation to HDMs, cockroaches, weed pollen, tree pollen and mould allergens over the past decade. However, the prevalence of pet allergens increased from 16.5% to 28.6%, showing a significant upward trend with an average annual rate increase of 1.3% (95% CI 0.85% to 1.67%, p<0.01; figure 2).

## Discussion

The production of sIgE is a hallmark of allergic sensitisation, and measurement of serum sIgE to common local aeroallergens has been widely utilised as a standard diagnostic tool for AR. In this study, we retrospectively analysed sIgE, testing results from 5486 outpatients with nasal hyper-reactivity symptoms during the past 10 years, in Guangzhou. This was in order to study the prevalence and trends of allergic sensitisation to common aeroallergens in Guangzhou.

The data from this study showed that HDMs were the predominant aeroallergen in Guangzhou, which was consistent with previously published studies.^7^ ^13^ High humidity and ambient temperatures have been reported as optimal conditions for HDM propagation.^14^ Guangzhou is located in a subtropical region with high temperature, high humidity and rainy weather, with little difference in perennial temperature. The characteristics of the local climate in Guangzhou are optimal for HDM propagation, which is in accordance with our results, with a high prevalence of sensitisation to HDMs observed in the region.

Lifestyle may be another potential contributor to the high prevalence of sensitisation to HDMs. In Guangzhou, residents in the municipality spend a majority of time indoors, especially in environments with air conditioning. The major sources of HDMs are bed sheets, carpets and pillows. A previous study confirmed the presence of high concentrations of *D. pteronyssinus* in air conditioning filters.^15^ Long durations in environments with poor ventilation increase the chance of exposure to HDMs.^14^ Another study showed that residing in homes with mechanical ventilation instead of air conditioning can reduce HDM exposure, leading to overall clinical improvement for HDM-sensitised patients.^16^

In this study, the prevalence of sensitisation to at least one aeroallergen was higher among the younger age groups (10–19 years). With every 10-year increase, the prevalence of sIgE decreased by 5.13%. These results suggest that sensitisation to allergens was most prevalent among younger patients, which was consistent with previously published studies.^17^ However, the exact reasons for the higher prevalence of sIgE observed in young patients with AR are unknown. One explanation may be immunosenescence, which reduces innate and adaptive immune responses, resulting in an attenuated response to foreign antigens and subsequently decreased sIgE production.^18^ ^19^ De Amici *et al*^20^ reported that the sIgE levels for several aeroallergens decreased significantly with increasing age. Further studies will be necessary to determine the impact of age on the positive rate of the serum sIgE test.

In this study, the prevalence of sensitisation to aeroallergens was within the range of 69.7–76.6%, from 2005 to 2014. We did not observe a significant change in sensitisation rates within the past 10 years, which was in accordance with studies performed by Jarvis *et al*.^21^ However, we did observe an upward trend in the sensitisation to pet allergens (dog hair and cat dander), rising from 16.5% to 28.6% at an average annual rate of 1.3%. These results indicate that over the past 10 years, an increasing number of patients with AR have become sensitised to pet allergens in Guangzhou.

The results from a cross-sectional questionnaire found that exposure to pets was a risk factor for respiratory diseases.^22^ Given the improvement in living standards in Guangzhou, an increasing number of people in the municipality keep cats and dogs as indoor pets, increasing the concentration of dog hair and cat dander, as well as the chance of exposure to these allergens. It was reported that long-term exposure to animal allergens may cause respiratory hyper-reactivity and accelerate the development of asthma and AR.^23^ The increasing prevalence of sensitisation to pet allergens within the past 10 years has indicated the importance of therapy for patients with AR with pet allergies. However, pet allergens were found not only in families with pets, but also in those without pets, and in public places, including communities and schools.^24^ Thus, the exact aeroallergen to be avoided was difficult to identify. Specific immunotherapy (SIT) is the only disease-modifying treatment for AR,^25^ and previous studies have reported that this treatment is clinically effective in desensitisation to pet allergens.^26^ ^27^ However, SIT for cat and dog allergens is still not available in China. Thus, this study further supports the importance of the introduction of pet SIT by Chinese health authorities and research institutions, allowing patients with AR and pets to cohabitate.

This is the first study on the trends in prevalence of common aeroallergen sensitisations to patients with AR over the last decade in Guangzhou. The study was based on the results of the serum sIgE test, a measurement with high specificity and sensitivity for the diagnosis of common aeroallergen sensitisation. However, there are several limitations in this study. The study was conducted by retrospective analysis of the results of serum sIgE testing. It was difficult to offer any information on the severity of AR symptoms and the risk factors for aeroallergen exposure. For example, although we found that an increasing number of patients with AR had been sensitised to pet allergens, we still have no details on pet keeping among those patients. Moreover, although all patients were from the majority of the districts in Guangzhou, this study was primarily a single-centre study. In addition, the sIgE results were obtained from the population of patients with nasal hypersensitivity, which may not be applicable to the general population in Guangzhou. From our results, we recommend that additional, multicentre studies be conducted to reach more accurate and generalised conclusions.

## Conclusion

This study demonstrated that HDMs comprise the most common aeroallergen in Guangzhou. The prevalence of sensitisation to aeroallergens decreased with increasing age. From 2005 to 2014, the prevalence of allergic sensitisation to pets has increased significantly in Guangzhou, suggesting the importance of introducing more effective measures for its prevention and treatment.

## References

[1] Alexandropoulos T, Haidich AB, Pilalas D Characteristics of patients with allergic rhinitis in an outpatient clinic: a retrospective study. Allergol Immunopathol (Madr) 2013;41:194–200. 10.1016/j.aller.2011.12.008 22405467

[2] Meltzer EO, Bukstein DA The economic impact of allergic rhinitis and current guidelines for treatment. Ann Allergy Asthma Immunol 2011;106:S12–16. 10.1016/j.anai.2010.10.014 21277528

[3] Okubo K, Kurono Y, Fujieda S Japanese guideline for allergic rhinitis 2014. Allergol Int 2014;63:357–75. 10.2332/allergolint.14-RAI-0768 28942929

[4] Patil VK, Kurukulaaratchy RJ, Venter C Changing prevalence of wheeze, rhinitis and allergic sensitisation in late childhood: findings from 2 isle of wight birth cohorts’ 12-years apart. Clin Exp Allergy 2015;45:1430–8. 10.1111/cea.12534 25809555

[5] Rönmark E, Bjerg A, Perzanowski M Major increase in allergic sensitization in schoolchildren from 1996 to 2006 in northern Sweden. J Allergy Clin Immunol 2009;124:357–63, 63.e1–15 10.1016/j.jaci.2009.05.011 19577282 PMC2747664

[6] Sakashita M, Hirota T, Harada M Prevalence of allergic rhinitis and sensitization to common aeroallergens in a Japanese population. Int Arch Allergy Immunol 2010;151:255–61. 10.1159/000242363 19786806

[7] Li J, Sun B, Huang Y A multicentre study assessing the prevalence of sensitizations in patients with asthma and/or rhinitis in China. Allergy 2009;64:1083–92. 10.1111/j.1398-9995.2009.01967.x 19210346

[8] Storkey J, Stratonovitch P, Chapman DS A process-based approach to predicting the effect of climate change on the distribution of an invasive allergenic plant in Europe. PLoS ONE 2014;9:e88156 10.1371/journal.pone.0088156 24533071 PMC3922760

[9] Van Bever HP, Lee BW, Shek LP Viewpoint: the future of research in pediatric allergy: what should the focus be? Pediatr Allergy Immunol 2012;23:5–10. 10.1111/j.1399-3038.2011.01245.x 22283402

[10] Baumann LM, Romero KM, Robinson CL Prevalence and risk factors for allergic rhinitis in two resource-limited settings in Peru with disparate degrees of urbanization. Clin Exp Allergy 2015;45:192–9. 10.1111/cea.12379 25059756 PMC5339878

[11] Mehulić M, Mehulić K, Vuljanko IM Changing pattern of sensitization in Croatia to aeroallergens in adult population referring to allergy clinic during a period of 15 years. Coll Antropol 2011;35:529–36. 21755728

[12] Bousquet J, Khaltaev N, Cruz A A Allergic Rhinitis and its Impact on Asthma (ARIA) 2008 update (in collaboration with the World Health Organization, GA(2)LEN and AllerGen). Allergy 2008,63(Suppl 86):8–160. 10.1111/j.1398-9995.2007.01620.x 18331513

[13] Zhang C, Li J, Lai X House dust mite and storage mite ige reactivity in allergic patients from Guangzhou, China. Asian Pac J Allergy Immunol 2012;30:294–300. 23393909

[14] Andiappan AK, Puan KJ, Lee B Allergic airway diseases in a tropical urban environment are driven by dominant mono-specific sensitization against house dust mites. Allergy 2014;69:501–9. 10.1111/all.12364 24456108 PMC4240470

[15] Zhang C, Gjesing B, Lai X Indoor allergen levels in Guangzhou city, southern China. Allergy 2011;66:186–91. 10.1111/j.1398-9995.2010.02465.x 20804467

[16] Harving H, Korsgaard J, Dahl R Clinical efficacy of reduction in house-dust mite exposure in specially designed, mechanically ventilated “healthy” homes. Allergy 1994;49:866–70. 10.1111/j.1398-9995.1994.tb00789.x 7709997

[17] Kato M, Yamada Y, Maruyama K Age at onset of asthma and allergen sensitization early in life. Allergol Int 2014;63(Suppl 1):23–8. 10.2332/allergolint.13-OA-0631 24809372

[18] Busse PJ, Mathur SK Age-related changes in immune function: effect on airway inflammation. J Allergy Clin Immunol 2010;126:690–9. 10.1016/j.jaci.2010.08.011 20920759 PMC3297963

[19] Ademokun A, Wu YC, Martin V Vaccination-induced changes in human b-cell repertoire and pneumococcal igm and iga antibody at different ages. Aging Cell 2011;10:922–30. 10.1111/j.1474-9726.2011.00732.x 21726404 PMC3264704

[20] De Amici M, Ciprandi G The age impact on serum total and allergen-specific ige. Allergy Asthma Immunol Res 2013;5:170 10.4168/aair.2013.5.3.170 23638316 PMC3636452

[21] Jarvis D, Luczynska C, Chinn S Change in prevalence of ige sensitization and mean total ige with age and cohort. J Allergy Clin Immunol 2005;116:675–82. 10.1016/j.jaci.2005.05.009 16159642

[22] Dong GH, Ma YN, Ding HL Pets keeping in home, parental atopy, asthma, and asthma-related symptoms in 12,910 elementary school children from northeast China. Indoor Air 2009;19:166–73. 10.1111/j.1600-0668.2008.00576.x 19076246

[23] Madhurantakam C, Nilsson OB, Uchtenhagen H Crystal structure of the dog lipocalin allergen can f 2: implications for cross-reactivity to the cat allergen fel d 4. J Mol Biol 2010;401:68–83. 10.1016/j.jmb.2010.05.043 20621650

[24] Kelly LA, Erwin EA, Platts-Mills TA The indoor air and asthma: the role of cat allergens. Curr Opin Pulm Med 2012;18:29–34. 10.1097/MCP.0b013e32834db10d 22081090 PMC3707607

[25] Pfaar O, Barth C, Jaschke C Sublingual allergen-specific immunotherapy adjuvanted with monophosphoryl lipid a: a phase i/iia study. Int Arch Allergy Immunol 2011;154:336–44. 10.1159/000321826 20975285

[26] Ling M, Long AA Pet dander and difficult-to-control asthma: therapeutic options. Allergy Asthma Proc 2010;31:385–91. 10.2500/aap.2010.31.3390 20929605

[27] Worm M, Lee HH, Kleine-Tebbe J Development and preliminary clinical evaluation of a peptide immunotherapy vaccine for cat allergy. J Allergy Clin Immun 2011;127:89–97, 97.e1–14 10.1016/j.jaci.2010.11.029 21211644

